# Profiles of social constraints and associated factors among breast cancer patients: a latent profile analysis

**DOI:** 10.1186/s12888-022-04407-y

**Published:** 2022-11-30

**Authors:** ChunYing Cui, Lie Wang, XiaoXi Wang

**Affiliations:** 1 Department of Social Medicine, School of Public Health, China Medical University, No.77 Puhe Road, Shenyang North New Area, 110122 Shenyang, Liaoning PR China; 2 Medical Basic Experimental Teaching Center, China Medical University, No.77 Puhe Road, Shenyang North New Area, 110122 Shenyang, Liaoning PR China

**Keywords:** cancer, Social constraints, Fear of progression, Social support, Latent profile analysis

## Abstract

**Background:**

The present study aimed to identify profiles of social constraints among Chinese breast cancer patients and to explore the variables associated with these patterns.

**Methods:**

The study recruited 133 Chinese breast cancer patients in Liaoning Province, China, between June 2021 and February 2022. The questionnaire package included the Social Constraints Scale (SCS), the Multidimensional Scale of Perceived Social Support (MSPSS), the Fear of Progression Questionnaire-Short Form (FoP-Q-SF), and the Social Impact Scale (SIS). The methods of statistical analysis used included latent profile analysis (LPA) and multinomial logistic regression.

**Results:**

Three latent patterns of social constraints were found: class 1-the low social constraints group (51.9%), class 2-the moderate social constraints group (35.3%), and class 3-the high social constraints group (12.8%). Patients with high social support were more likely to report a low level of social constraint, while patients with a greater fear of progression were more likely to report a moderate or high level of social constraints. Significant differences existed among the latent classes identified by reference to social constraint in terms of education.

**Conclusion:**

These results suggest that breast cancer patients’ perceptions of social constraints vary and exhibit individual differences. Health care providers should take into account patients’ fear of progression as well as their social support when developing interventions for patients with a high level of social constraints.

## Introduction

Social constraints are defined as referring to both the objective existence and subjective perceptions of social conditions that prevent individuals from disclosing their illness-related feelings or concerns or modify the mode of such disclosure[1]. According to social cognitive processing theory (SCPT) [2], the suppression of social emotions can hinder the individual’s opportunities to make sense of his or her cancer experience and cognitive processes, which can result in psychological disorders and intrusive thoughts regarding cancer [3, 4]. In addition, social constraints can cause the individual to avoid thinking and talking about cancer to maintain interpersonal harmony [3, 4]. As a result, social constraint responses from the individual’s family and friends may result in self-stigmatization due to the feeling that cancer is “bad luck” and places a burden on the family [5], which leads to persistent psychological distress [6], impaired quality of life [7], worse psychological adjustment [3] and higher rates of posttraumatic stress disorder (PTSD) symptoms [8].

Breast cancer surpassed lung cancer as the leading type of cancer worldwide in 2020 [9]. Breast cancer is considered to be a life-threatening event and can often lead to impaired functioning and bodily disfigurement. Breast cancer patients who experience negative feelings and thoughts related to their cancer experience are more likely to express their concerns regarding cancer [10]. However, their partners, family members, or friends may not be ready to discuss these topics or may respond in a socially constrained way to minimize their fear and discomfort [1, 10]. In addition, when cancer patients want to discuss their fears, their partners, family members or friends may complain [10]. Given these problems, cancer patients cannot express their thoughts and feelings concerning the recurrence of cancer freely. As a result, they may process their fear inadequately [2] and experience elevated self-doubt in the context of coping with cancer recurrence [11], which in turn increases their psychological distress. Emerging research has studied the adverse influence of social constraints on higher levels of psychological adjustment [6, 12–14], PTSD [8, 15–17], fear of recurrence [18, 19], and poor sleep quality [20–22] and quality of life [7, 23] among cancer patients. Our study, therefore, posits that social constraints might play a significant role in psychological adjustment in this context.

However, previous studies have mainly focused on the associations among social constraints, psychological health problems and quality of life among cancer patients, and few such studies have explored the patterns of social constraints among cancer patients or associated factors. In addition, previous studies concerning social constraints have often used variable-focused analytical techniques, which presuppose that the psychological status of patients is distributed homogeneously. However, other studies have demonstrated that this distribution is heterogeneous in the wake of trauma (e.g., cancer diagnosis), suggesting that research that employs variable-focused analytical techniques might be unable to reflect the psychological responses associated with patients’ heterogeneity since it neglects individual differences [24, 25]. Therefore, a person-focused analytical technique was used to explore patterns of social constraints among breast cancer patients.

Latent profile analysis (LPA) is a person-focused analysis approach used to identify individuals according to similar features and to classify similar individuals into latent discrete groups. The LPA results should indicate that breast cancer patients in the same latent group are homogeneous while breast cancer patients in different latent groups are heterogeneous in terms of social constraints. Lanza et al. also claimed that LPA is an ideal method for exploring social relationship profiles [26]. Therefore, the present study aimed to identify the profiles of social constraints associated with Chinese breast cancer patients by using LPA as well as to explore the sociodemographic and clinical characteristics related to these profiles. In addition, this study examined the associations between these profiles and patients’ fear of progression, self-stigma, and social support.

## Methods

### Participants

The present study was conducted in Liaoning Province, China, from June 2021 to February 2022. All participants were recruited at the affiliated Hospital of China Medical University. The inclusion criteria for the current study required participants to be over 18 years old, informed of their cancer diagnosis (breast cancer), able to communicate and read well in Chinese. The exclusion criteria for this study included patients with other severe diseases (such as severe cardiovascular disease, a history of psychiatric treatment, or cognitive and intellectual disorders). Self-report questionnaires were distributed to each eligible patient after receiving their written informed consent for participation in this study. Ultimately, 133 of 165 breast cancer patients effectively completed the survey, for an effective response rate of 80.6%. Twenty-three patients refused to participate this investigation, and nine questionnaires were excluded due to invalid data (missing data > 20%). Therefore, 133 breast cancer patients participated in the survey. The study was approved by the Ethics Committee of the First Affiliated Hospital of China Medical University (NO. 2021-430-2), and all participants provided written informed consent prior to completing the survey.

### Measures

#### Social constraints

The 15-item Social Constraints Scale (SCS-15) was originally developed by Lepore and Ituarte [27]. The original scale contains 15 items, and each item is scored on a four-point scale ranging from 1 (never) to 4 (often). A higher score indicates a higher frequency of experiencing social constraints. Two items (“tell you not to worry so much about your health” and “tell you to try not to think about cancer”) were deleted due to their low item-total correlations (*r* = 0.33–0.36) compared to other items (*r* = 0.53–0.72). In a previous study, Yeung et al. [20] also excluded these two items due to low inter-item correlations and item-total correlations in the context of Chinese-American breast cancer patients and claimed that the 13-item version largely retained the factors that were measured by the original scale. Therefore, our study analysed 13 items, including “Changed the subject”, “Did not understand your mood/situation”, “Avoided you”, “Trivialized your problems”, “Hid feelings”, “Acted uncomfortably”, “Minimized your problems”, “Complained about own problems”, “Acted cheerful around you”, “Did not want to hear about your illness”, “Felt uncomfortable and made you keep feelings to yourself”, “Felt upset and made you keep feelings to yourself”, and “Did not show concern as you expected”. Copyright authorization for the use of the SCS was obtained from Lepore, the developer of the original scale. In addition, our study obtained copyright authorization for the use of the Chinese language SCS from You and Lu [7]. The Chinese version of the scale has been used to investigate Chinese breast cancer patients [28]. In addition, to ensure the reliability and stability of the factor structure of the 13-item version, a supplementary confirmatory factor analysis (CFA) was conducted. The goodness-of-fit for the 13-item social constraints scale based on a single dimension model was χ^2^/df = 1.821, AGFI = 0.830, NFI = 0.901, TLI = 0.932, CFI = 0.952, IFI = 0.953, RMSEA = 0.079, which indicated satisfactory model fit. The Cronbach’s coefficient alpha (*α*) for the scale was 0.909.

### Social support

The Multidimensional Scale of Perceived Social Support (MSPSS) [29] was used to test the levels of social support exhibited by Chinese breast cancer patients. The MSPSS includes 12 items, and each item is scored on a 7-point scale ranging from 1 (very strongly disagree) to 7 (very strongly agree). A higher score indicates a higher level of social support. The Chinese version of the scale has been used widely to investigate Chinese cancer patients [30, 31]. The Cronbach’s alpha coefficient for the MSPSS was 0.965 in the current research.

### Fear of progression

The 12-item short version of the Fear of Progression Questionnaire-Short Form (FoP-Q-SF) [32] was used to investigate the levels of fear of progression exhibited by Chinese breast cancer patients. The FoP-Q-SF comprises 12 items, and each item is scored on a 5-point scale ranging from 1 (never) to 5 (very often). The Chinese version of the scale has been shown to have good reliability and validity in the context of investigating other cancer patients [33]. A higher score indicates a more severe fear of progression. The Cronbach’s alpha coefficient for the FoP-Q-SF was 0.897 in the present study.

### Self-stigma

The Chinese version of the Social Impact Scale (SIS) [34] was used to measure the levels of stigma experienced by Chinese breast cancer patients in the present study. Each item included in the SIS is scored on a 4-point Likert-type scale ranging from “1 = very disagree” to “4 = very agree”; higher scores indicate higher levels of stigma. This scale has been widely used to investigate Chinese cancer patients [30]. The Cronbach’s alpha coefficient for the SIS was 0.956 in the present study.

### Demographic and clinical characteristics

Demographic data were collected, including age, residence, marital status, educational background, monthly family income (CNY), current levels of smoking and drinking, religious faith, and children. Clinical data were collected, including cancer diagnosis, time since diagnosis, and distant metastasis.

### Statistical methods

First, descriptive statistics were used to describe the variables measured in terms of frequency, percentage, mean, and SD.

Second, to determine the optimal class solution, a series of LPA models with an increasing number of latent classes were developed using Mplus software (1–5 classes). Several fit indicators were used to evaluate the quality of the different models. Lower Akaike information criterion (AIC), Bayesian information criterion (BIC), adjusted Bayesian information criterion (aBIC), and higher entropy values (> 0.8) indicated better model fit [35]. Additionally, the Lo-Mendell-Rubin (LMR) and the Bootstrap Likelihood Ratio Test (BLRT) were used to compare the solution with k classes and the solution with k-1 classes, and the statistically significant *p* values suggested an improvement in fit due to the inclusion of an additional class [36]. In addition, Nylund et al. demonstrated that BLRT is the most consistent indicator of classes across all the models considered, followed by BIC [37].

Third, the chi-square test and analysis of variance (ANOVA) techniques were used to determine whether all measured variables made distinctions among classes. A chi-square test was used to identify the demographic and clinical characteristics that could be used distinguished the classes. Analysis of variance (ANOVA) was conduct to assess the differences among the continuous variables (age, social support, self-stigma, and fear of progression). Finally, multinomial logistic regression was performed to identify the factors that predicted different profiles of social constraints. Mplus version 8.3 software was used to conduct LPA, and SPSS version 20.0 software was used to conduct the other statistical analyses. A two-tailed *P* < 0.05 was considered to be statistically meaningful.

## Results

### Characteristics of the participants

Among the 133 participants in this study, the mean age was 48.09 (SD = 10.20). The detailed demographic characteristics of these participants are presented in Table 1. Regarding cancer diagnosis (Table 1), more than one-fifth of participants reported a time after diagnosis of more than two years, and 52.6% of participants had distant metastasis. Breast cancer patients were diagnosed at stage I (36.1%) and stage II (63.9%).

**Table 1:** Distribution of demographic/clinical data

Variables	Frequency (*n*)	Percent (*%*)
Residence		
Rural area	30	22.6
Urban area	103	77.4
Marital status		
Single/divorced/widowed/separated	22	16.5
Married/cohabited	111	83.5
Education		
Junior school or lower	44	33.1
High school	34	25.6
Junior college	28	21.1
College or higher	27	20.3
Family per capita monthly income		
< 3000	57	42.9
3000–5000	41	30.8
≥ 5000	35	26.3
Smoking		
No	123	92.5
Yes	10	7.5
Drinking		
No	104	78.2
Yes	29	21.8
Religious faith		
No	123	92.5
Yes	10	7.5
Children		
No	10	7.5
Yes	123	92.5
Time since diagnosis		
Half year or below	47	35.3
Half to 2 years	57	42.9
More than 2 years	29	21.8
Distant metastasis		
No	63	47.4
Yes	70	52.6
Cancer stage		
I	48	36.1
II	85	63.9

### Latent profile analysis

Fit indices of the five LPA models are presented in Table 2. The five-class model had the lowest AIC, BIC, and aBIC values, and these indices decreased with an increasing number of classes. The entropy values of all models were above 0.9, suggesting that all models facilitated accurate classification. The BLRT values of all models were significant. However, the two- and three-class pattern models had more significant LMR values than the four- and five-class pattern models, suggesting that the four- and five-class patterns should be excluded. BLRT proved to be the most consistent indicator of classes among all of the models considered, followed by BIC. The BIC value of the three-class models was lower than that of the two-class models. Therefore, the three-class patterns were shown to be optimal in the present study.

**Table 2:** Latent class model fit comparison

Models	AIC	BIC	aBIC	Entropy	LMR	BLRT
1-class	4709.076	4784.225	4701.984			
2-class	4151.744	4267.358	4140.833	0.966	0.023	< 0.001
3-class	3984.595	4140.673	3969.865	0.963	0.046	< 0.001
4-class	3837.367	4069.910	3854.818	0.965	0.130	< 0.001
5-class	3806.472	4043.480	3784.104	0.985	0.586	< 0.001

*AIC *Akaike information criterion, *BIC *Bayesian information criterion, *aBIC *adjusted Bayesian information criterion, *LMR *Lo-Mendell-Rubin, *BLRT *Bootstrap likelihood ratio test

**Fig. 1:**
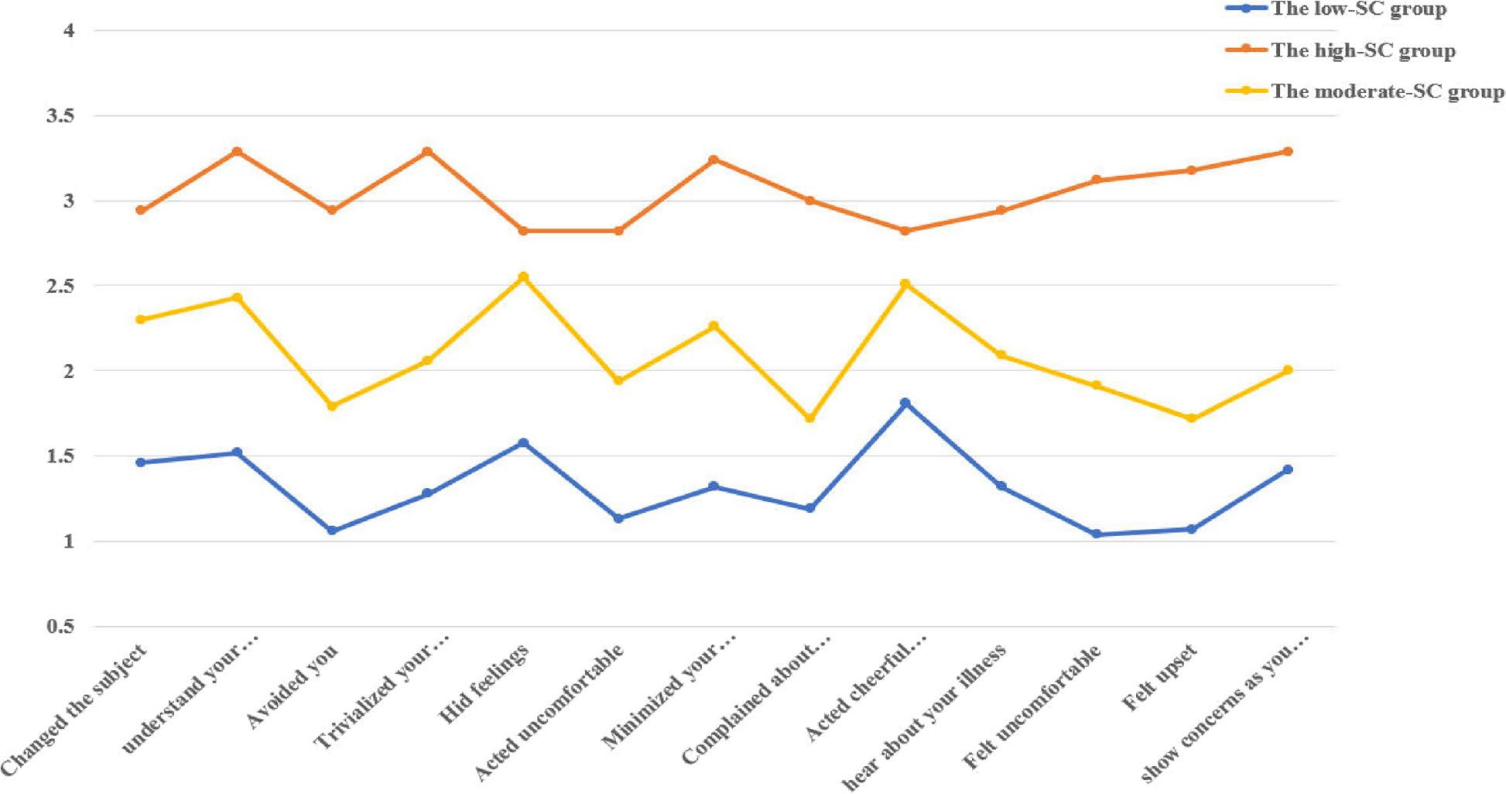
Latent profile plot based on the social constraints (SC) for breast cancer patients

Three profiles of social constraints are depicted in Fig. 1. Class 1 (*n* = 69, 51.9%) was characterized by the lowest level of social constraints (SCS total score mean = 17.20, SD = 3.30). Therefore, class 1 was labelled the “low social constraints group”. Class 2 (*n* = 47, 35.3%) was characterized by a moderate level of social constraints (SCS total score mean = 27.28, SD = 3.07) and was identified as the “moderate social constraints group”. Class 3 (*n* = 17, 12.8%) was characterized by the highest level of social constraints (SCS total score mean = 39.71, SD = 4.81) and was named the “high social constraints group”.

### Multinomial logistic regression analysis across the identified latent classes

As shown by the univariate analysis (Tables 3 and 4), significant differences existed among the latent classes identified by social constraints in terms of education, fear of progression, self-stigma, and social support. Based on these results, multinomial logistic regression was conducted using the potential categories as dependent variables and the significant factors in the univariate analysis as independent variables. Table 5 illustrates the results of the multiple logistic regression analysis of these factors, thus identifying different patterns of social constraints. Compared to the group with low social constraints, breast cancer patients with a junior school or lower level of education were more likely to report a high level of social constraints (OR = 8.898, 95% CI = 1.127–70.265); breast cancer patients with lower levels of social support were more likely to report a moderate level of social constraints (OR = 0.967, 95% CI = 0.940–0.995) or a high level of social constraints (OR = 0.929, 95% CI = 0.884–0.976); and breast cancer patients with greater fear of progression were more likely to report a moderate level of social constraints (OR = 1.101, 95% CI = 1.031–1.175) or a high level of social constraints (OR = 1.175, 95% CI = 1.048–1.318).

**Table 3:** Differences in demographic and clinical characteristics and continuous variables among the latent classes (n, %)

Variables	The low- SC group (*n* = 69) (*%*)	The moderate-SC group (*n* = 47) (*%*)	The high- SC group (*n* = 17) (*%*)	*χ* ^2^	*P*-value
Residence				0.501	0.779
Rural area	15 (21.7)	10 (21.3)	5 (29.4)		
Urban area	54 (78.3)	37 (78.7)	12 (70.6)		
Marital status				0.547	0.761
Single/divorced/widowed/separated	11 (15.9)	9 (19.1)	2 (11.8)		
Married/cohabited	58 (84.1)	38 (80.9)	15 (88.2)		
Education				16.113	0.013
Junior school or lower	17 (24.6)	15 (31.9)	12 (70.6)		
High school	17 (24.6)	16 (34.0)	1 (5.9)		
Junior college	19 (27.5)	7 (14.9)	2 (11.8)		
College or higher	16 (23.2)	9 (19.1)	2 (11.8)		
Monthly family income				6.889	0.142
< 3000	26 (37.7)	21 (44.7)	10 (58.8)		
3000–5000	20 (29.0)	15 (31.9)	6 (35.3)		
≥ 5000	23 (33.3)	11 (23.4)	1 (5.9)		
Smoking				4.664	0.097
No	67 (97.1)	41 (87.2)	15 (88.2)		
Yes	2 (2.9)	6 (12.8)	2 (11.8)		
Drinking				1.302	0.522
No	53 (76.8)	36 (76.6)	15 (88.2)		
Yes	16 (23.2)	11 (23.4)	2 (11.8)		
Religious faith				1.340	0.512
No	104 (92.9)	55 (96.5)	19 (90.5)		
Yes	8 (7.1)	2 (3.5)	2 (9.5)		
Children				4.190	0.123
No	8 (11.6)	1 (2.1)	1 (5.9)		
Yes	61 (88.4)	46 (97.9)	16 (94.1)		
Time since diagnosis				1.853	0.763
Half year or below	23 (33.3)	16 (34.0)	8 (47.1)		
Half to 2 years	32 (46.4)	20 (42.6)	5 (29.4)		
More than 2 years	14 (20.3)	11 (23.4)	4 (23.5)		
Distant metastasis				0.889	0.641
No	30 (43.5)	24 (51.1)	9 (52.9)		
Yes	39 (56.5)	23 (48.9)	8 (47.1)		
Cancer stage				0.516	0.773
I	23 (33.3)	18 (38.3)	7 (41.2)		
II	46 (66.7)	29 (61.7)	10 (58.8)		

*SC S*ocial constraints

**Table 4:** Comparison of continuous variables between the low-, moderate -, and high-SC groups

Variables	The low-SC group	The moderate-SC group	The high-SC group	*F*	*P*-value
Age	46.32±10.14	50.89±10.10	47.53±9.53	2.922	0.057
Fear of progression	13.74±8.80	19.91±6.33	24.06±7.34	16.078	< 0.001
Self-stigma	44.01±12.96	50.82±10.63	57.82±10.63	9.956	< 0.001
Social support	65.33±15.15	58.70±14.38	50.41±15.29	7.768	0.001

*SC *Social constraints

**Table 5:** Factors in differentiating distinct social constraints groups

Variables	The moderate-SC group			The high-SC group		
	OR	95%CI	*P*	OR	95%CI	*P*
Education						
Junior school or lower	1.926	0.581–6.381	0.284	8.898	1.127–70.265	0.038
High school	1.897	0.581–6.195	0.289	0.726	0.045–11.751	0.821
Junior college	0.738	0.198–2.748	0.651	1.399	0.123–15.962	0.787
College or higher	Ref			Ref		
Fear of progression	1.101	1.031–1.175	0.004	1.175	1.048–1.318	0.006
Self-stigma	1.006	0.966–1.048	0.758	1.049	0.976–1.127	0.196
Social support	0.967	0.940–0.995	0.023	0.929	0.884–0.976	0.003

SC, social constraints

Reference group is the low-SC group

Nagelkerke R^2^ = 0.422

## Discussion

To our knowledge, our research is the first study to use the LPA technique to identify specific patterns of social constraints in breast cancer patients. Additionally, the present study aimed to identify group differences in demographic and clinical characteristics, fear of progression, self-stigma, and social support across the identified latent classes.

### Profiles of social constraints

The LPA method is commonly used to identify latent characteristics in diverse populations. Cai et al. [38] reported that three profiles of social relationships were identified in Chinese breast cancer patients undergoing chemotherapy and emphasized the significance of developing tailored interventions for individuals in the high-risk group. In addition, Shim et al. [39] categorized breast cancer patients based on their depression symptoms using the LPA approach and suggested that targeted psychological interventions should be conducted based on the specifics of different classes. The results of the current study indicated three latent patterns of social constraints: class 1-the low social constraints group (51.9%), class 2-the moderate social constraints group (35.3%), and class 3-the high social constraints group (12.8%). These results suggested that breast cancer patients’ perceptions of social constraints varied and exhibited individual differences.

### Predictors of latent class membership

Our study investigated the predictors of specific patterns of social constraints and found that the identified classes of breast cancer patients exhibited significant differences in terms of their level of education. Breast cancer patients with lower levels of education were more likely to belong to the high social constraints group. To our understanding, education serves as a proxy for psychological resources, including knowledge, intelligence, cognitive resources, and the ability to manage disease [40]. In the specific context of cancer, education is associated with a better understanding of cancer, higher health literacy, and greater ability to manage symptoms and make medical decisions [41]. Furthermore, previous studies have reported that education is positively associated with social support [23] and that cancer patients with higher social support tend to perceive lower social constraints [42].

Moreover, our study found that fear of progression (FoP) is more likely to be associated with the groups associated with moderate and high social constraints, thus suggesting that fear of progression might be a crucial risk factor with respect to social constraints among breast cancer patients. FoP refers to patients’ fear that the illness and all its biopsychosocial consequences will progress or recur in the same part or another part of the body [43]. Such fear is based on the personal experience of an incapacitating or life-threatening illness and becomes manifest in cognitive, behavioural, emotional, and physiological qualities [43]. Fear focuses on patients’ perceived threats to the self or losses, which can motivate an individual’s negative cognition [44]. In addition, an increase in a patient’s level of fear of cancer progression is related to a decrease in the patient’s physical activities [45] and may harm the patient’s positive social relationships, which can lead to social constraints.

Furthermore, our study found that patients with higher levels of social support are less likely to be categorized into the groups with moderate or high social constraints. Based on the stress-buffering hypothesis [46], individuals’ perceived stressors can become less harmful as a result of perceived social support. Responses to Social constraints from cancer patients’ networks (e.g., denial, withdrawal, and criticism of the patients’ disclosure) can weaken the patient’s evaluation of their social relationships and their perceived control over their disease [1]. Therefore, a supportive social network (e.g., network interactions that involve sharing dinner or expressing affection) may mitigate the negative implications of social constraints. Lepore et al. [42] and Chu et al. [17] also reported similar findings in the contexts of prostate cancer and breast cancer, respectively.

### Implications

According to our results, health care providers can identify breast cancer patients who face greater risks from issues pertaining to social constraints. Health care providers should pay more attention to negative social network relationships, such as denial, withdrawal, and criticism of the patients’ disclosure. The findings of the present study indicated that emotional concealment and behavioural camouflage (e.g., situations in which others acting cheerful around the patient to hide their real feelings or worries regarding the patient or when they feel upset and cause the patient to keep his or her feelings to himself or herself) are common kinds of social constraints. Therefore, intervention strategies that focus on personal disclosure should be adopted. However, Chinese people are reluctant to express their feelings and thoughts regarding cancer diagnosis and treatments due to cultural perceptions [47]. Thus, expressive writing is a culturally appropriate intervention for Chinese cancer patients because it addresses their need for emotional expression and is well suited to their cultural values, which emphasize the suppression of emotions in public [48]. For instance, Chu et al. [16] found that expressive writing interventions effectively contribute to mitigating the impact of social constraints and decreasing posttraumatic stress disorder (PTSD) among Chinese American BCP. In addition, it is crucial to consider the patient’s level of education, fear of progression, and social support. For example, sufficient information and support regarding cancer diagnosis and treatments should be provided to patients with low levels of education, and their fear of progression should be decreased. Practitioners and caregivers must identify patients’ needs for various types of social support to offer emotional, informational and affectionate support [17].

### Limitations

The present study faced several limitations. First, the self-report survey used in our study entails inherent bias. In addition, the current study was conducted in one institution in Liaoning Province, China, which may limit its generalizability. Second, our study included a variety of types of breast cancer and did not differentiate patients according to cancer type. Therefore, future studies should conduct further investigations to investigate specific types of cancer. Finally, the number of participants included in the study was relatively small; thus, future research should be conducted by reference to a larger sample to reexamine our conclusions.

## Conclusion

Our study was the first to identify specific patterns of social constraints in breast cancer patients by using the LPA technique. Our findings indicated three latent patterns of social constraints, i.e., class 1-the low social constraints group; class 2-the moderate social constraints group; and class 3-the high social constraints group, which were significantly associated with education, fear of progression, and social support. Intervention strategies focusing on social constraints should thus take the fear of progression and social support into account.

## Data Availability

The dataset in this study is available from the corresponding author upon reasonable request.

## Abbreviations

FoP-Q-SF: The fear of progression questionnaire-short form
LPA: Latent profile analysis
SCS: The social constraints scale
SIS: The social impact scale
AIC: Akaike information criterion
BIC: Bayesian information criterion
aBIC: adjusted bayesian information criterion
LMR: Lo-mendell-rubin
BLRT: Bootstrap likelihood ratio test
GFI: Goodness of fit index
AGFI: Adjusted goodness of fit index
NFI: Normed fit index
TLI: Tucker Lewis index
RMSEA: Root mean square error of approximation
ANOVA: Analysis of variance
